# All-Trans Retinoic Acid plus Arsenic Trioxide versus All-Trans Retinoic Acid plus Chemotherapy for Newly Diagnosed Acute Promyelocytic Leukemia: A Meta-Analysis

**DOI:** 10.1371/journal.pone.0158760

**Published:** 2016-07-08

**Authors:** Yafang Ma, Lu Liu, Jie Jin, Yinjun Lou

**Affiliations:** 1 Institute of Hematology, Department of Hematology, The First Affiliated Hospital, Zhejiang University, Hangzhou, China; 2 Key Laboratory of Hematopoietic Malignancies Zhejiang Province, Hangzhou, China; Queen's University Belfast, UNITED KINGDOM

## Abstract

**Background:**

Recently, the all-trans retinoic acid (ATRA) plus arsenic trioxide (ATO) protocol has become a promising first-line therapeutic approach in patients with newly diagnosed acute promyelocytic leukemia (APL), but its benefits compared with standard ATRA plus chemotherapy regimen needs to be proven. Herein, we conducted a meta-analysis comparing the efficacy of ATRA plus ATO with ATRA plus chemotherapy for adult patients with newly diagnosed APL.

**Methods:**

We systematically searched biomedical electronic databases and conference proceedings through February 2016. Two reviewers independently assessed all studies for relevance and validity.

**Results:**

Overall, three studies were eligible for inclusion in this meta-analysis, which included a total of 585 patients, with 317 in ATRA plus ATO group and 268 in ATRA plus chemotherapy group. Compared with patients who received ATRA and chemotherapy, patients who received ATRA plus ATO had a significantly better event-free survival (hazard ratio [HR] = 0.38, 95% confidence interval [CI]: 0.22–0.67, p = 0.009), overall survival (HR = 0.44, 95% CI: 0.24–0.82, p = 0.009), complete remission rate (relative risk [RR] = 1.05; 95% CI: 1.01–1.10; p = 0.03). There were no significant differences in early mortality (RR = 0.48; 95% CI: 0.22–1.05; p = 0.07).

**Conclusion:**

Thus, this analysis indicated that ATRA plus ATO protocol may be preferred to standard ATRA plus chemotherapy protocol, particularly in low-to-intermediate risk APL patients. Further larger trials were needed to provide more evidence in high-risk APL patients.

## Introduction

Acute promyelocytic leukemia (APL) was a specific type of acute myeloid leukemia (AML) associated with the fusion of promyelocytic (PML) gene with the retinoic acid receptorα(RARA) gene (PML-RARA) generated by the t(15;17) translocation [1–3]. Historically, APL was highly fatal due to early bleeding complications [4, 5]. In 1988, the Shanghai group first reported the use of differentiation-induction therapy with all-trans retinoic acid (ATRA) in APL patients [6]. Over the past three decades, there has been a revolutionary improvement in the outcomes for patients with APL [7]. Currently, with the all-trans retinoic acid (ATRA) combined to chemotherapy treatment, more than 85% of patients with APL are curable in the context of multicenter clinical trials [8–10]. Thus, much focus has been placed on decreasing early mortality, reducing relapse rate, minimizing toxicity and improving the quality of life [11–13].

In addition to ATRA, arsenic trioxide (ATO) subsequently has been found with high efficacy in relapsed APL patients [14–16]. Several latest trials have provided vital data on efficacy and safety of ATRA plus ATO with or without chemotherapy protocol for first-line therapy in newly diagnosed APL patients [17–23]. However, the comparative effectiveness of these treatment strategies with classic ATRA plus chemotherapy has not been well systematically evaluated. The best first-line therapy for APL patients remains to be determined.

Thus, this meta-analysis aims to comparatively evaluate the efficacy of ATRA plus ATO and ATRA plus chemotherapy in newly diagnosed adult APL patients with the intention of providing assistance in clinical decision-making.

## Methods

### Literature search and identification of eligible trials

The English databases including PubMed, the Web of Science, the Cochrane Library and the abstracts of the American Society of Hematology were searched for all studies comparing ATRA plus ATO combination therapy with ATRA plus chemotherapy. The search terms used were “arsenic trioxide” or “ATO”, or “all-trans-retinoic acid” or “ATRA”, and “acute promyelocytic leukemia”, “APL” or “M3” from their inception to February 18, 2016, without restriction of languages. The reference lists of the included articles were also screened for potential studies.

Studies were eligible for inclusion in the meta-analysis if they conformed to prospective trials that compared ATRA plus ATO with ATRA plus chemotherapy in induction and consolidation therapy in newly diagnosed APL patients. The exclusion criteria were: (i) the same population included in several studies or duplicate articles; (ii) retrospective research; (iii) only children <16 years included; (iv) the study didn’t provide complete data to be statistically analyzed.

### Data extraction and methodological quality assessment

Two authors (YFM and LL) independently searched and screened the studies according to the criteria mentioned above, and then extracted data from included studies. Disagreements were resolved by reaching a consensus with another author (YJL). Incomplete data was sought from the authors. Basic characteristics of the trials, patients, and outcomes were extracted.

### Statistical analysis

Dichotomous outcomes were pooled to obtain a relative risk (RR). Time to event (survival-type) data was analyzed by the log hazard ratio (HR) and its standard error (SE). Both of them had a 95% confidence interval (CI). Log HR and its SE were calculated according to HR (including its CI), p-value, survival curves and/or observed number of events provided in the trial publications [24, 25]. Statistical homogeneity among studies was assessed using the Cochran Q test and I^2^ statistic. Data were pooled by fixed-effects models if no significant heterogeneity was found (Q test P≥0.10 or I^2^≤50%) [26]. Otherwise, random-effects models were chosen. Sensitivity analysis was scheduled to perform comparisons according to original risk stratification of patients, the variation of intervention measures and different types of studies. Forest plots were used to present the results of the meta-analysis while the funnel plot was performed to assess the publication bias. A P value <0.05 was considered to be statistically significant. Review Manager 5.3 (RevMan 5.3®, Nordic Cochrane Center and Copenhagen, Denmark) was used to perform the statistical analysis.

## Results

### Selection of the trials

**Fig 1:**
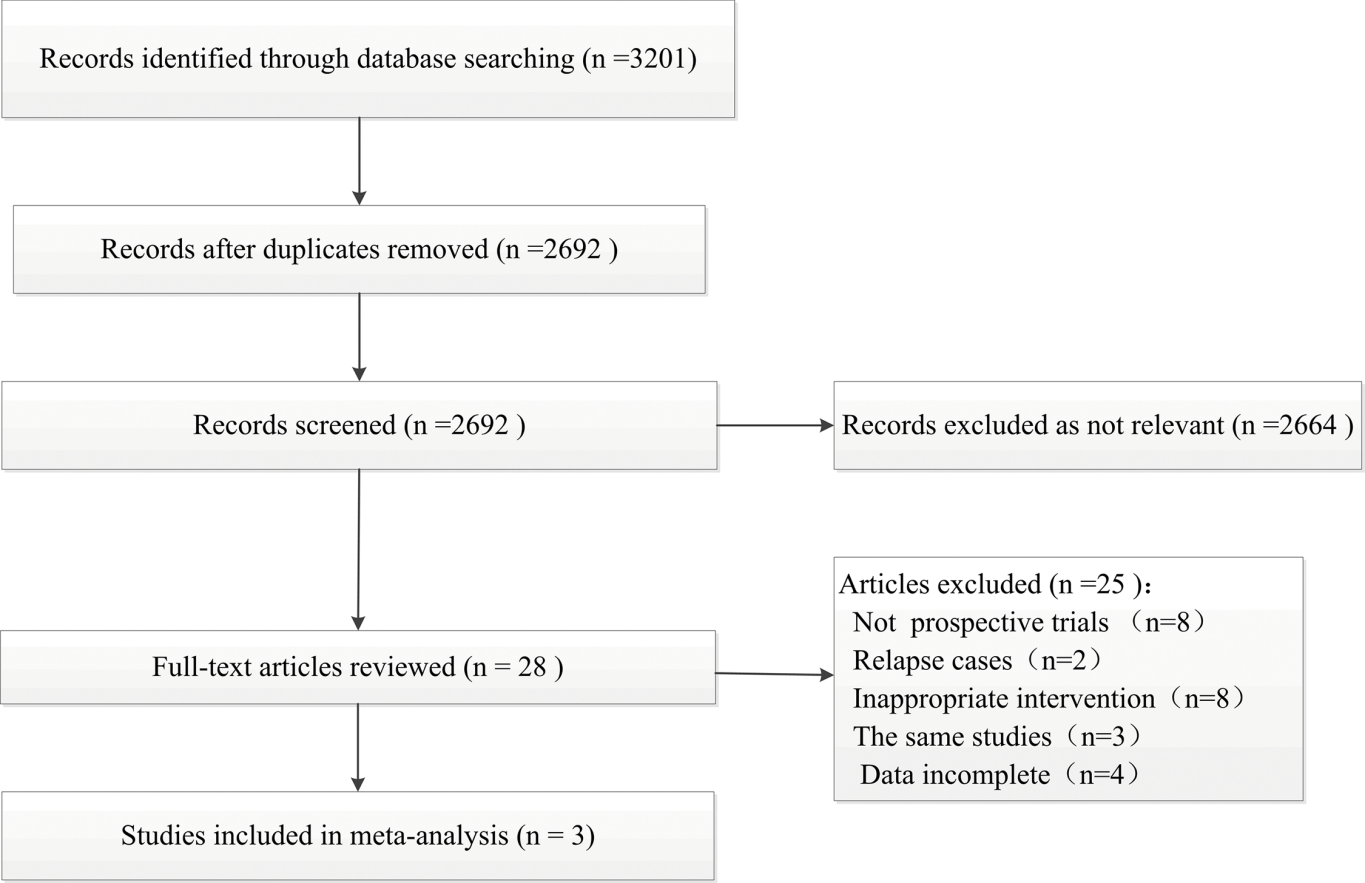
Flow diagram of selection of studies for conducting meta-analysis.

Based on the pre-defined search strategy, 3201 potentially relevant trials were found from the primary retrieval. After the process of advanced retrieval (Fig 1), three trials involving 585 patients, were identified for inclusion in the meta-analysis.

### Description of studies and regimens

The general characteristics of the included studies are shown in Table 1. The trial results were published between 2013 and 2015. Two trials (APL0406 [the Italian-German APL 0406 trial] and AML17 [the UK NCRI AML17 Clinical Trial for APL]) were randomized controlled trials by comparing ATRA plus ATO with ATRA plus chemotherapy [17, 18]. Both of the trials described the methods of randomization, allocation concealment and using an intention-to-treat analysis [17, 18]. The APML4 (Australasian Leukemia and Lymphoma Group APML4 trial) study was an excellent prospective multicenter trial although the history APML3 data were used as the control. No trial reported blinding of participants, personnel or the progress of outcome assessment. The quality of these studies was high, as assessed with the method of the Cochrane Collaboration.

**Table 1:** Characteristics of included Studies.

Study (Author,publication year)	Lo-Coco et al 2013	Burnett et al 2015	Iland et al 2015
**Protocol**	APL0406	AML17 for APL	APML4
**Rondomization**	Yes	Yes	No
**No. patients(Male/Female)**	156	235	194
** ATRA plus ATO group**	40/37	60/56	62/62
** ATRA plus chemotherapy group**	36/43	60/59	37/33
**Median age(range)**			
** ATRA plus ATO group**	44.6(19.1–70.2)	47(16–75)	44(3–78)
** ATRA plus chemotherapy group**	46.6(18.7–70.2)	47(16–77)	39(19–73)
**Sanz risk**			
** Low-to-intermediate**	156	178	156
** High risk**	NA	57	38
**Induction regimen**			
** ATRA plus ATO group**	ATRA plus ATO	ATRA plus ATO±GO	ATRA plus ATO and IDA
** ATRA plus chemotherapy group**	AIDA schedule^a^	AIDA schedule	AIDA schedule
**Consolidation therapy**			
** ATRA plus ATO group**	ATRA plus ATO 4 cycles	ATRA plus ATO 4 cycles	ATRA plus ATO 2 cycles
** ATRA plus chemotherapy group**	AIDA schedule	AIDA schedule	AIDA schedule
**Maintenance therapy**			
** ATRA plus ATO group**	No	No	ATRA/MTX/6-MP for 2 years
** ATRA plus chemotherapy group**	ATRA/MTX/6-MP for 2 years	No	ATRA/MTX/6-MP for 2 years
**Median follow up (months)**	34.4	30.5	50.4
**Cumulative ATO days**	112 days^b^	63 days	81 days
**Cumulative ATO doses**	16.8mg/kg^b^	17.0mg/kg	12.2mg/kg

ATRA, all-trans retinoic acid; ATO arsenic trioxide; IDA, idarubicin; MTX, methotrexate; 6-MP, 6-mercaptopurine; GO, gemtuzumab ozogamicin.

^a^ AIDA schedule:According to the standard all-trans retinoic acid and idarubicin based therapy.

^b^ Based on the median time to complete remission.

One trial (APL0406) only enrolled the low-to-intermediate risk patients, while the other two trials (AML17 and APML4) enrolled both low-to-intermediate risk and high-risk patients. Also, it is important to point out that the chemo-free ATRA plus ATO approach was used for the low-to-intermediate-risk APL subgroup in two trials (APL0406 and AML17) [17, 18]. The AML17 trial added gemtuzumab ozogamicin during induction therapy for high-risk APL subgroup. The Australasian APML4 trial combined ATRA, ATO and idarubicin during induction, followed by consolidation with ATO plus ATRA without chemotherapy. The cumulative ATO doses in three trials were 16.8, 17.0, and 12.2mg/kg in APL0406, AML17, and APML4, respectively. The median follow-up time was 30.5–50.4 months in the three trials (Table 1).

### Complete remission (CR)

**Fig 2:**
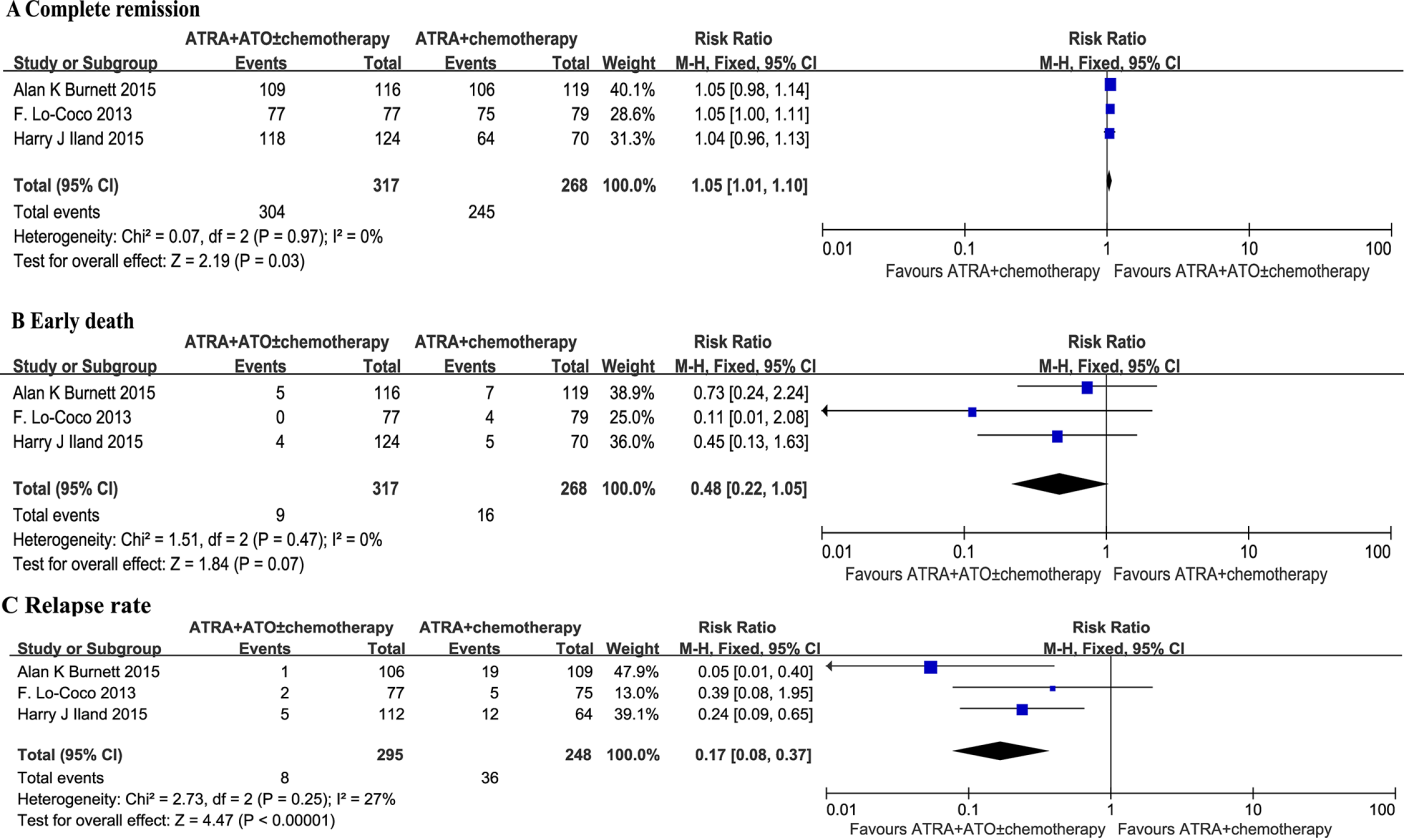
Forest plot of findings of (A) Complete remission, (B) Early death, (C) Relapse rate between patients receiving ATRA plus ATO versus ATRA plus chemotherapy.

All three studies investigated the rate of CR. Initial baseline characteristics between the treatment group and the control group were quite balanced. It was observed that there was no statistical heterogeneity among the trials found (p = 0.97; I^2^ = 0%). With the fixed effects model, there was a statistically significant increase in CR rate in patients given ATRA plus ATO regimens compared to patients that received conventional ATRA plus chemotherapy protocols (RR = 1.05; 95% CI: 1.01–1.10; p = 0.03) (Fig 2A).

### Early death

All three studies describe the rate of early death. No statistical heterogeneity among the studies was found (p = 0.47; I^2^ = 0%). With the fixed effects model there was no significant difference between the two groups (RR = 0.48; 95% CI: 0.22–1.05; p = 0.07) (Fig 2B).

### Relapse rate

The fixed effects model was used as no statistical heterogeneity among the studies was found (p = 0.25; I^2^ = 27%). There was a statistically significant reduction in relapse rate in the ATRA plus ATO group compared with the standard ATRA plus chemotherapy group (RR = 0.17, 95% CI: 0.08–0.37, p < 0.00001) (Fig 2C).

### Relapse-free survival (RFS)

**Fig 3:**
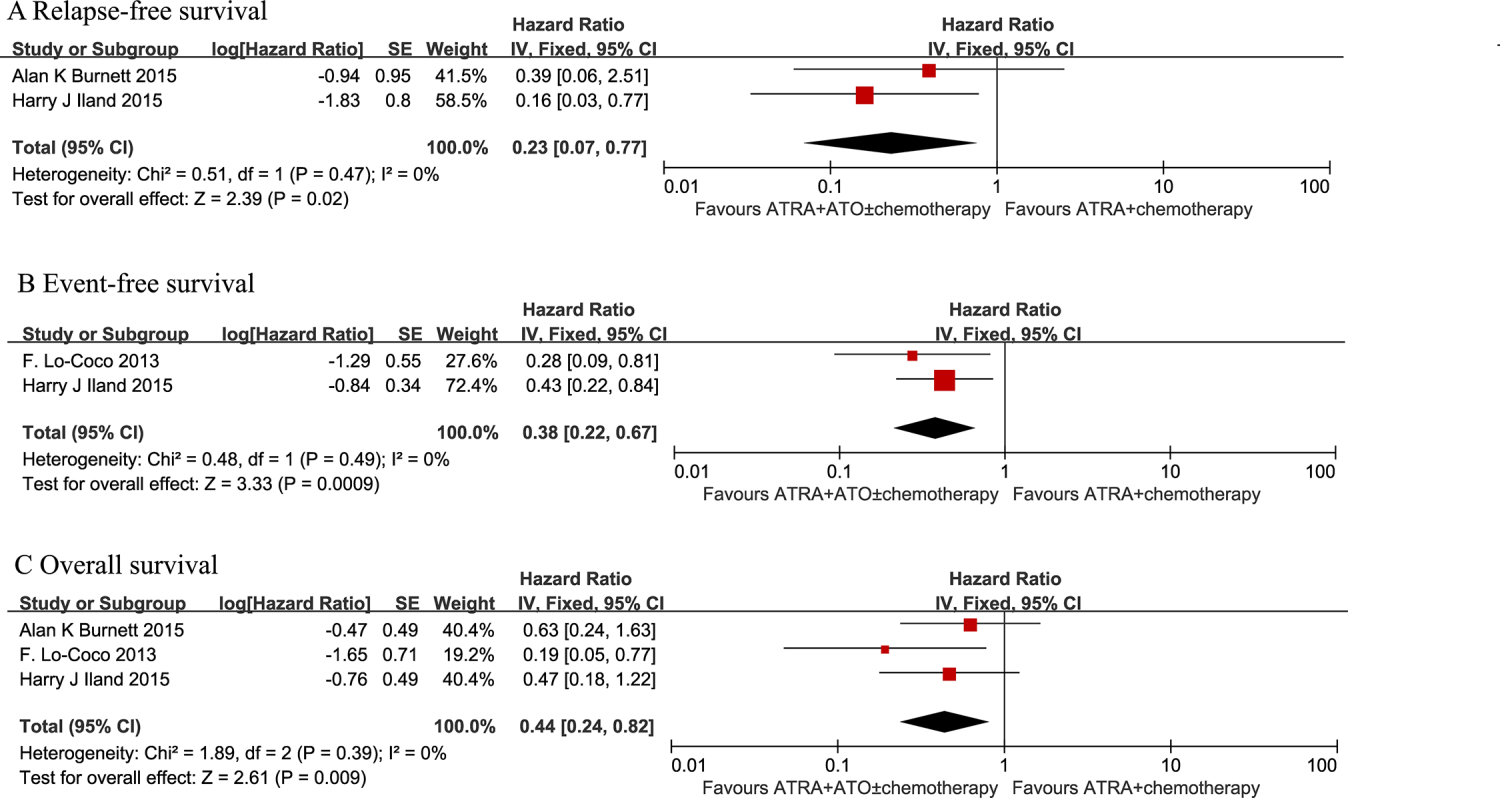
Forest plot of findings of (A) Relapse-free survival, (B) Event-free survival, (C) Overall survival between patients receiving ATRA plus ATO versus ATRA plus chemotherapy.

Two studies (AML17 and APML4) evaluated RFS. As expected, patients in the ATRA plus ATO group had a significantly better RFS compared to patients in the ATRA plus chemotherapy group in a fixed-effects model. (HR = 0.23, 95% CI: 0.07–0.77, p = 0.02) (Fig 3A).

### Event-free survival (EFS)

Two studies (APL0406 and APML4) evaluated EFS. No significant heterogeneity (p = 0.49; I^2^ = 0%) was observed between the results obtained in the included studies, so an analysis was performed with a fixed effects model. There was a statistically significant increase in EFS in patients treated with ATRA plus ATO regimens when compared to patients given conventional ATRA plus chemotherapy regimens at 2 years (HR = 0.38, 95% CI: 0.22–0.67, p = 0.009) (Fig 3B).

### Overall survival (OS)

All three studies evaluated OS. The median observation time ranged from 30 to 50 months, and the number of patients for OS was calculated by assuming a 2-year OS from published studies. No significant heterogeneity (p = 0.39; I^2^ = 0%) was observed among the included studies. Therefore, an analysis was performed with a fixed-effects model. Patients in the ATRA plus ATO group had a statistically significant better OS compared with the ATRA plus chemotherapy group (HR = 0.44, 95% CI: 0.24–0.82, p = 0.009) (Fig 3C).

## Discussion

This meta-analysis of three published comparative studies, reporting results of 585 adults patients with APL showed a significant improvement in EFS, RFS and OS in patients given ATRA plus ATO compared with patients given conventional ATRA plus chemotherapy. Also, the ATRA plus ATO significantly decreased the relapse rate and adverse events. Of note, there was no statistical difference in early death between the two groups.

Two previous meta-analyses assessed the efficacy of ATO for APL patients [27, 28]. Both analyses suggested that ATRA and ATO combination therapy may be effective for the treatment of APL. However, the meta-analysis by Wang et al in 2011 evaluated two randomized trials with a small population and three with non-randomized data [28]. In 2012, Chen et al. analyzed six non-randomized studies [27]. Importantly, the control groups in both previous meta-analyses were not the standard ATRA plus chemotherapy schedule. Our meta-analysis combined the results of two larger randomized studies and one well designed prospectively trial with history control. All the control groups were current standard ATRA plus chemotherapy regimens. Thus, the high quality of the trials, in general, allowed us to believe that the conclusions are comparatively reliable.

The synergism effects between ATRA and ATO has been demonstrated to eradicate APL stem cells through PML-RARA degradation [29]. Interestingly, as early as 2006, Estey, et al. initially reported the chemo-free ATRA plus ATO regimen as an alternative to chemotherapy in newly diagnosed APL [23]. Furthermore, the chemo-free protocol of APL0406, and the minimized chemotherapy schedule in the AML17 and APML4 trials in this meta-analysis confirmed that chemotherapy may safely be omitted in low-to-intermediate risk patients. These studies not only translated the cooperative effect of ATRA plus ATO from bench into clinic practice but also provided a paradigm of a synergistic targeting therapy model in the leukemia field.

Although the majority of the patients in these trials are in low-to-intermediate risk patients, both the AML17 and APML4 trials included a small number of high-risk APL patients. It should be pointed out that the high-risk subgroup patients in these two trials were treated identically to low-to-intermediate risk patients except during induction. These results suggest that the high-risk APL patients may also benefit from the ATRA plus ATO therapy. In our experience, if high-risk patients can survive initial induction, the introduction of ATO in first-line therapy may provide excellent RFS and OS [30]. However, further larger prospective randomized trials are needed to confirm the potential benefits of ATO in high-risk APL.

In the ATRA era, maintenance therapy appears to be useful in APL [31]. There was no maintenance therapy in the ATRA plus ATO arm during the APL0406 and APML17 trial. These trials suggested that for in patients receiving ATO therapy, maintenance treatment may not be necessary.

In addition to the efficacy, safety is an equally important issue. Two trials (APL0406 and AML17) compared toxicity between the two groups. One trial (APML4) only described adverse events in the ATRA plus ATO group. Overall, neutropenia and thrombocytopenia were significantly less frequent in the ATRA plus ATO group. The hepatic toxic effects and prolongation of the QTc interval were manageable. Moreover, the APL0406 trial showed that Health-related quality of life was significantly improved in the ATRA plus ATO treatment arm compared to that of the ATRA plus chemotherapy arm [13]. In the AML17 trial, although there was no statistically significant difference in quality of life between the ATRA plus ATO and ATRA plus chemotherapy arms, there was significantly less supportive care in the ATRA plus ATO group. In our experience, the incidence of secondary leukemia or secondary primary malignancies is also rare.

However, several limitations should be kept in mind. First, a relatively small number of studies were included with one of them being a non-randomized trial. Second, there was heterogeneity in the types of ATRA plus ATO regimens administered among the trials. The optimal doses and scheme for ATO in APL treatment remain uncertain. Third, the adverse effects were recorded inconsistently among the three trials. However, all studies suggested an acceptable toxicity profile of ATRA plus ATO therapy.

Despite these limitations, our meta-analysis demonstrated a significant benefit of ATRA plus ATO protocol as compared to ATRA plus chemotherapy protocol. Therefore, we believe that the administration of ATRA plus ATO should be considered a more appealing option in adult patients with newly diagnosed APL. In the future, large randomized control trial and long-term follow-up data are needed to provide more evidence in high-risk APL patients.

## Supporting Information

S1 Checklist PRISMA Checklist. (DOC)
